# Ultrasound-guided caudal anaesthesia combined with epidural anaesthesia for caesarean section: a randomized controlled clinical trial

**DOI:** 10.1186/s12884-024-06298-1

**Published:** 2024-02-02

**Authors:** Fangjun Wang, Qi Lü, Min Wang, Hongchun Xu, Dan Xie, Zheng Yang, Qin Ye

**Affiliations:** 1 Department of Anesthesiology, Affiliated Hospital, North Sichuan Medical College, No. 63, Cultural Road, Shunqing District, NanchongCity, Sichuan Province China; 2 Department of Operation Center, Affiliated Hospital, North Sichuan Medical College, Nanchong, 637000 China; 3 North Sichuan Medical College, Nanchong, 637000 China

**Keywords:** Caudal anaesthesia, Epidural anaesthesia, Spinal anaesthesia, Caesarean section

## Abstract

**Background:**

Although epidural anaesthesia and spinal anaesthesia are currently the general choices for patients undergoing caesarean section, these two neuraxial anaesthesia methods still have drawbacks. Caudal anaesthesia has been considered to be more appropriate for gynaecological surgery. The purpose of this study was to compare epidural anaesthesia combined with caudal anaesthesia, spinal anaesthesia and single-space epidural anaesthesia for caesarean section with respect to postoperative comfort and intraoperative anaesthesia quality.

**Methods:**

In this clinical trial, 150 patients undergoing elective caesarean section were recruited and randomized into three groups according to a ratio of 1:1:1to receive epidural anaesthesia only, spinal anaesthesia only or epidural anaesthesia combined with caudal anaesthesia. The primary outcome was postoperative comfort in the three groups. Secondary outcomes included intraoperative anaesthesia quality and the incidences of nausea, vomiting, postdural puncture headache, maternal bradycardia, or hypotension.

**Results:**

More patients were satisfied with the intraoperative anaesthesia quality in the EAC group than in the EA group (*P* = 0.001). The obstetrician was more significantly satisfied with the intraoperative anaesthesia quality in the SA and EAC groups than in the EA group (*P* = 0.004 and 0.020, respectively). The parturients felt more comfortable after surgery in the EA and EAC groups (*P* = 0.007). The incidence of maternal hypotension during caesarean section was higher in the SA group than in the EA and EAC groups (*P* = 0.001 and 0.019, respectively).

**Conclusions:**

Epidural anaesthesia combined with caudal anaesthesia may be a better choice for elective caesarean section. Compared with epidural anaesthesia and spinal anaesthesia, it has a higher quality of postoperative comfort and intraoperative anaesthesia.

## Introduction

In obstetric anaesthesia, anaesthesiologists must choose an anaesthesia method that is safe and comfortable for the mother, as well as a method that has little effect on the newborn and that can provide obstetricians with good surgical conditions. Epidural or spinal anaesthesia is increasingly used in caesarean section because it is safer for foetuses and pregnant women than general anaesthesia [1]. During neuraxial anaesthesia, adverse reactions are very rare, and contact between the newborn and mother can be established immediately after delivery.

Although spinal anaesthesia is more popular in parturients undergoing caesarean section (due to its shorter anaesthetic time and ease of operation) [2], it has some significant complications, such as postdural puncture headache (PDPH), extensive block and hypotension [3, 4]. During spinal anaesthesia, a denser motor block than epidural anaesthesia can lead to prolonged numbness and paralysis of the lower limbs during and after the operation [5]. Due to the deficiencies in spinal anaesthesia, epidural anaesthesia may be the best choice for parturients undergoing caesarean section [1]. However, the incidence of unsatisfactory anaesthesia that requires intervention is relatively high during epidural anaesthesia for caesareansection [6]. All of the deficiencies observed during spinal anaesthesia and epidural anaesthesia may result in decreased intraoperative and postoperative comfort in parturients.

During caesarean section, the abdominal skin incision is located between the T_10_ and T_12_ dermatomes, and the surgical operation is mainly performed in the pelvic cavity. Therefore, a caesarean section anaesthetic segment requires a distance from at least the 10th thoracic nerve to the sacral nerves, and cephalad block sensory level up to T_4_ is needed for caesarean section in a clinical aspect [7]. Epidural puncture is generally performed at the L_2–3_ intervertebral space during epidural anaesthesia for caesarean section [4]. Single-space epidural anaesthesia has difficulty achieving such extensive nerve blocking, and the epidural administration of a large volume of high-concentration local anaesthetic may cause systemic toxicity. For reliable sacral nerve block, caudal anaesthesia (used as a supplementary mode of analgesia) has been considered to be more appropriate for gynaecological surgery, and many studies have shown that the application of ultrasound has increased the safety, ease, and consistency of caudal analgesia [8–11]. Thus, we speculated that epidural anaesthesia within the T_11_-T_12_ intervertebral space with high-concentration local anaesthetics combined with caudal anaesthesia with high-volume and low-concentration local anaesthetics could achieve satisfactory anaesthesia for caesarean section with less numbness in the lower extremities and more stable maternal haemodynamics. However, no experiments have yet been conducted with respect to the effects of epidural anaesthesia combined with caudal anaesthesia for caesarean section.

In the present study, we aimed to compare the postoperative comfort quality and intraoperative anesthesia quality of patients receiving single point epidural anesthesia, spinal anesthesia, and epidural anesthesia combined with caudal anesthesia.

## Methods

Following approval by the ethics committee, we obtained written informed consent from all of the participants for this clinical trial. This prospective, randomized controlled study was registered prior to patient enrolment at the Chinese Clinical Trial Registry (Registration number: ChiCTR-INR-16008933, Date: 28/07/2016).

From August 2016 to May 2017, 159 parturients at term were assessed for eligibility in this study. All of the participants were primigravida, American Society of Anaesthesiologists (ASA) grade II, single birth, and undergoing optional caesarean section. Exclusion criteria included a history of hypersensitivity to the drugs used, contraindications for regional block (such as infection of the puncture site, anatomic deformities, or coagulation disorders), diagnosis of acute or chronic foetal distress, prior administration of opioids and other central nervous system depressants, rhesus immunization, pregnancy-induced hypertension syndrome, body mass index (BMI) > 35 kg/m^2^, and intraoperative blood loss> 800 ml. The patients were randomized into three groups by using a computer-generated list of random numbers, and sealed envelopes were used to receive Single-space epidural anaesthesia only (EA group), spinal anaesthesia only (SA group), or epidural anaesthesia combined with caudal anaesthesia (EAC group).

The day before surgery, the study protocol, including epidural anaesthesia, spinal anaesthesia and epidural anaesthesia combined with caudal anaesthesia procedures, was explained to each parturient. All parturients were made familiar with the use of a numeric rating scale (NRS) identifying 1 as unsatisfactory and 5 as very satisfied [12]. All of the intrathecal blocks isolated from obstetrician were performed by the same anaesthesiologist who was proficient in neuraxial anaesthesia and had more than 10 years of experience in anaesthesia. The caesarean section of all parturients in this study was performed by a deputy chief obstetrician.

The epidural puncture sites were at the L2–3 intervertebral space in the EA and SA groups [4] and the T11-T12intervertebral space in the EAC group. The epidural space was localized and confirmed with the loss of resistance to air technique. The intrathecal injection of 3 ml 0.5% ropivacaine was performed in the SA group by the needle-through-needle technique, and then an epidural catheter was inserted 4.5 cm into the epidural space in a cephalic direction and gently withdrawing either no cerebrospinal fluid or blood in all groups. In the EAC group, a 22-gauge needle was inserted into the sacral hiatus using ultrasound to guide accurate placement of the needle. After prior negative blood aspiration, a caudal injection of 0.225% ropivacaine 20 ml administered in >30s was performed, and any untowards effect was observed for 5–10 minutes. After the epidural catheter was secured to the skin surface, the participants were repositioned with left uterine displacement by keeping a wedge beneath the right half of the lower back, and a pillow was placed below the head and shoulders. Thereafter, 3 ml of 2% lidocaine hydrochloride solution was administered as a test dose, and any unintended effect was observed in the EA and EAC groups. After 5–8 minutes of administration of the test dose, the EA group and EAC group received epidural anaesthesia with 16 mL and 10 mL of 0.75% ropivacaine, respectively. Surgical procedures were initiated only after the dermatomes blocked were completely established until T10 or 20 min had passed after completion of spinal or epidural anaesthetic. A modified Bromage motor scale (MBS) (1 = Complete block, unable to move feet or knees; 2 = Almost complete block, able to move feet only; 3 = Partial block, just able to move knees; 4 = Detectable weakness of hip flexion while supine, full flexion of knees; and 5 = No detectable weakness of hip flexion while supine) [13] was used to assess motor block. When the parturient felt pain or discomfort during the operation, 1 mg of midazolam and 20 mg of ketamine were administered intravenously [14]. If caesarean section could not be performed after intravenous analgesic administration, neuraxial anaesthesia was converted to general anaesthesia. The following variables were recorded: time to initial onset of cryanaesthesia at T10, maximal sensory blockade segments, time to attain maximum motor blockade, and time for complete regression of motor block. Maternal haemodynamic parameters, including NIBP (both systolic and diastolic), ECG, heart rate, SpO2and respiratory rate, were monitored continuously, and recordings were made every 1 minute until 30 minutes after the local anaesthetic was administered and at 5-minute intervals thereafter up to the end of surgery. Hypotension (defined as systolic falling more than 20% before anaesthesia or systolic values lower than 80 mmHg) was treated with an ephedrine 6 mg intravenous bolus immediately. Bradycardia (defined as heart < 55 beats/minute) was treated with 0.5 mg of injection atropine.

During the surgical procedure, the number of parturients who complained of pain or discomfort, the administered dosage of midazolam and ketamine, and the number of patients in which neuraxial anaesthesia was converted to general anaesthesia in each group were recorded. Intraoperative adverse events such as nausea, vomiting, postdural puncture headache, maternal bradycardia or hypotension were recorded. Nausea and/or vomiting were treated with 4 mg ondansetron intravenously. At the end of surgery, the anaesthesia quality was assessed by the parturient and the obstetrician on a numeric rating scale (NRS) from 1(not satisfied at all) to 5 (very satisfied) [12]. All of the patients returned to the maternity ward and received epidural analgesia after caesarean section. To avoid PDPH, patients in the SA group were placed in a supine position for at least 4 hours after surgery. The quality of postoperative comfort was evaluated by the parturient 12 h after operation on a numeric rating scale (NRS) (1 = Not satisfied at all; 2 = Unsatisfied; 3 = Less satisfied; 4 = Satisfied; 5 = Very satisfied) [12]. When the score of anaesthesia quality or postoperative comfort was less than 4, it was defined as patient dissatisfaction. Thus, patients who score above or equal to 4 are considered satisfied, and those who score below 4 are regarded as unsatisfied. The satisfaction rate is equal to the number of parturients with an NRS greater than or equal to 4 divided by the total number of parturients in each group. All indicators were assessed and recorded by a research assistant who was unaware of the grouping of clinical trials.

## Statistical analysis

A comparison of the quality of postoperative comfort of patients was the primary outcome of this study. In the preliminary experiment, we found that the postoperative satisfaction rates of patients in the spinal anaesthesia group, epidural anaesthesia group and epidural anaesthesia combined with caudal anaesthesia group were 53, 75 and 85%, respectively. We calculated that a sample size of 49 patients would be needed in each group (type I error of 0.05, power of 0.9). Considering a 10% dropout rate, 53 patients in each group were necessary for analysis. The following formula was used to compute the sample size:

$$n=\frac{1641.6\lambda }{{\left({\mathit{\sin}}^{-1}\sqrt{Pmax}-{\mathit{\sin}}^{-1}\sqrt{Pmin}\right)}^2}$$

The statistical software SPSS (version 19.0) was used for all of the statistical analyses. The one-sample Kolmogorov–Smirnov test was applied to analyse the distribution of the data, and each group of data was subjected to a homogeneity test for variance in multiple samples by the means of Levene’s test. One-way analysis of variance was used to examine the differences in parturient demographic data, the duration of surgery, and the time to complete regression of motor block between groups. The nonnormal distribution of measurement data, such as maximal sensory blockade spinal segments, maximum motor block scores, the time to cryanaesthesia at T_10_, the time to maximum motor block, and analgesic and sedative drug usage, were analysed by the Kruskal–Wallis H(K) test. The incidences of maternal bradycardia, hypotension, nausea, vomiting, postoperative headache, pain or discomfort, conversion from neuraxial anaesthesia to general anaesthesia, quality of anaesthesia and postoperative comfort among the three groups were compared with the chi-square test or Fisher’s exact test. A *p* value < 0.05 was considered to be statistically significant.

## Results

**Fig. 1:**
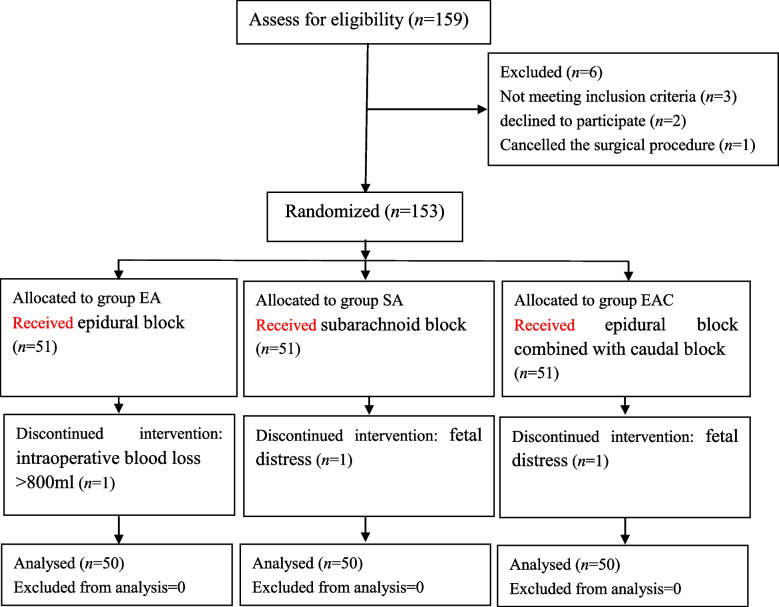
Study flow diagram

A total of 159parturients were enrolled in this study between August 2016 and May2017, of whom 6 were excluded; in total, 153 parturients were randomized into three groups according to the ratio of 1:1:1. Later, one parturient was excluded for intraoperative blood loss> 800 ml in the EA group, and one parturient was excluded for foetal distress in the SA group and EAC group. Thus, 50 parturients in each group were analysed for this study (shown in Fig. 1).

Parturient demographic data are summarized in Table 1. There were no significant differences among the three groups regarding age, height, weight, or gestational age (*p* = 0.307, 0.319, 0.852, and 0.427, respectively).

**Table 1:** Characteristics of participants for the three groups

Variables	EA group *n* = 50	SA group *n* = 50	EAC group *n* = 50	*F values*	*P values*
Age (year)	27.8 ± 5.5	26.5 ± 3.2	27.3 ± 4.0	1.190	0.307
Height (cm)	161.4 ± 6.7	160.2 ± 5.4	159.5 ± 6.3	1.151	0.319
Weight (kg)	67.8 ± 9.5	68.2 ± 9.8	67.1 ± 10.3	0.161	0.852
Gestational age(d)	274.4 ± 8.4	271.6 ± 9.6	272.3 ± 9.8	0.856	0.427

Values are presented as mean ± SD. *EA* epidural anaesthesia, *SA* spinal anaesthesia, *EAC* epidural anaesthesia combined with caudal anaesthesia

The quality of postoperative comfort and intraoperative anaesthesia are shown in Tables 2 and 3. The postoperative satisfaction rates were 76, 56, and 84% in the EA, SA, and EAC groups, respectively. The parturients felt more comfortable after surgery in the EA and EAC groups than in the SA group (*P* = 0.016 and 0.001, respectively). The intraoperative satisfaction rates were 64, 70, and 86% in the EA group, SA group, and EAC group, respectively. Patients were more significantly satisfied with the intraoperative anaesthesia quality in the EAC group than in the EA group (*P* = 0.001). The obstetrician was more significantly satisfied with the intraoperative anaesthesia quality in the SA and EAC groups than in the EA group (*P* = 0.004 and 0.020, respectively).

**Table 2:** The quality of postoperative comfort

Groups	*n*	1	2	3	4	5
EA^*^	50	0	1	11	10	28
SA	50	0	4	18	15	13
EAC ^*^	50	0	0	8	12	30
*χ*^2^	18.063					
*P*	0.003					

Values are number of patients. *EA* epidural anaesthesia, *SA* spinal anaesthesia, *EAC* epidural anaesthesia combined with caudal anaesthesia. ^*^ *P* < 0.05 vs. SA group

**Table 3:** The anesthesia quality was judged by the parturients and the obstetrician

Groups	*n*	Parturients					Obstetrician				
		1	2	3	4	5	1	2	3	4	5
EA*	50	3	3	12	20	12	1	3	12	16	18
SA*^#^	50	0	0	15	15	20	0	0	4	12	34
EAC ^#^	50	0	0	7	13	30	0	0	6	12	32
*χ*^2^		20.349					16.675				
*p*		0.002					0.011				

Values are number of patients. *EA* epidural anaesthesia, *SA* spinal anaesthesia, *EAC* epidural anaesthesia combined with caudal anaesthesia. The anesthesia quality was judged by the parturients, *P < 0.05 vs. EAC group; the anesthesia quality was judged by the obstetrician, ^#^*P* < 0.05 vs. EA group

The initial block characteristics are shown in Table 4.Compared with the EA and EAC groups, the time to cryanaesthesia at T_10_ was shorter in the SA group (*p* < 0.001). The maximum sensory blockade segments were increased more significantly in the SA and EAC groups than in the EA group (*p* < 0.001). Compared with the EA and EAC groups, the maximum motor block scores were lower in the SA group (*p* < 0.001), and the maximum motor block scores were increased more significantly in the EAC group than in the EA and SA groups (*p* < 0.001). The time to maximum motor block was shorter in the SA group than in the EA and EAC groups (*p* < 0.001), and the time to complete regression of motor block was longer in the SA group than in the EA and EAC groups (*p* < 0.001). The time for complete regression of the motor block in the EAC group was shorter than that in the EA group (*p* = 0.032).

**Table 4:** Initial block characteristics

Variables	EA group *n* = 50	SA group *n* = 50	EAC group *n* = 50	*F values*	*P values*
Time to cryanaesthesia at T_10_(minutes), median (IQR)	13.5(10.5–17)^*^	6(5–6)	12(10–14)^*^	67.071	.000
Maximal Sensory blockade segments, median (IQR)	11(9–12)	15(15–16)^#^	15(14–16)^#^	96.829	.000
Maximum motor block scores, median (IQR)	1(1–2)^*^	1(1–1)	2(1–2)^*#^	29. 592	.000
Time to maximum motor block (minutes), median (IQR)	15.5(13–18)^*^	8(7–9)	14.5(13–16)^*^	67.786	.000
Time for complete regression of motor block (minutes), mean ± SD	160.9 ± 12.6^*^	190.0 ± 13.2	150.0 ± 13.1^*#^	127.090	.000

Values are presented as mean ± SD or median (IQR). *EA* epidural anaesthesia, *SA* spinal anaesthesia, *EAC* epidural anaesthesia combined with caudal anaesthesia. ^*^P < 0.001 vs. SA group, ^#^*P* < 0.05 vs. EA group

Information regarding intraoperative pain or discomfort, analgesic and sedative drug usage, conversion of neuraxial anaesthesia to general anaesthesia, and duration of surgery is shown in Table 5. Epidural anaesthesia was converted to general anaesthesia in six parturients in the EA group, whereas no parturient in which neuraxial anaesthesia was converted to general anaesthesia in the SA and EAC groups. The incidence of conversion from neuraxial anaesthesia to general anaesthesia was higher in the EA group than in the SA and EAC groups (*P* = 0.012). Eighteen parturients (36%) in the EA group, 15 parturients (30%) in the SA group, and seven parturients (14%) in the EAC group experienced pain or discomfort during surgery. The incidence of pain or discomfort was higher in the EA group than in the EAC group (*P* = 0.001). Compared with the EA group, the dosage of midazolam administered was lower in the EAC group (*P* = 0.004), and the dosage of ketamine administered was lower in the SA group (*P* = 0.024) and EAC group (*P* = 0.004). Compared with the EA group, the duration of surgery was shorter in the SA and EAC groups (*p* = 0.001 and 0.007, respectively). The incidence of postoperative numbness and motor weakness in the lower extremities was lower in the EAC group (*p* = 0.041).

**Table 5:** Information regarding intraoperative pain or discomfort, analgesic and sedative drugs usage, conversion of neuraxial anesthesia to general anesthesia, duration of surgery, and postoperative numbness and motor weakness in lower extremities

Variables	EA group *n* = 50	SA group *n* = 50	EAC group *n* = 50	*F/ χ*^*2*^ *values*	*P values*
Intraoperative pain or discomfort, n (%)	18(36)	15(30)	7(14) ^*^	6.614	0.037
Midazolam dosage (mg), median(IQR)	0(0–1)	0(0–1)	0(0–0) ^*^	7.810	0.020
Ketamine dosage (mg), median (IQR)	0(0–20)	0(0–0) ^*^	0(0–0) ^*^	8.204	0.017
Conversion of neuraxial anesthesia to general anesthesia, n (%)	6(12)	0(0) ^*^	0(0) ^*^	9.722	0.003
Duration of surgery (minutes), mean ± SD	63.2 ± 9.0	56.9 ± 9.6^*^	57.9 ± 10.2 ^*^	6.246	0.002
Postoperative numbness and motor weakness, n (%)	10(20)	11(22)	3(6)^*^	6.328	0.041

*EA* epidural anesthesia for cesarean section, *SA* spinal anesthesia, *EAC* epidural anesthesia combined with caudal anesthesia. ^*^P < 0.05 vs. EA group

The side effects are shown in Table 6. The incidence of maternal hypotension was higher in the SA group than in the EA and EAC groups (*P* = 0.001 and 0.019, respectively). There was no significant difference among the three groups regarding maternal bradycardia, nausea, vomiting and postdural puncture headache (PDPH) (*P* = 0.773, 0.613, 0.469 and 0.232, respectively).

**Table 6:** Side effects

Variables	EA *n* = 50	SA *n* = 50	EAC *n* = 50	*χ*^2^ *values*	*P values*
Maternal bradycardia, *n* (%)	1(2)	2(4)	1(2)	0.514	0.773
Maternal hypotension, *n* (%)	10(20) ^*^	22(44)	11(22) ^*^	8.672	0.013
Nausea, *n* (%)	5(10)	7(14)	4(8)	0.979	0.613
Vomiting, *n* (%)	3(6)	4(8)	2(4)	1.515	0.469
Postdural puncture headache, *n* (%)	1(2)	4(8)	1(2)	2.924	0.232

Values are number of patients (%). *EA* epidural anesthesia, *SA* spinal anesthesia, *EAC* epidural anesthesia combined with caudal anesthesia, ^*^P < 0.05 vs. SA group

## Discussion

The current study showed that parturients felt more comfortable after surgery in the EA and EAC groups, whereas patients in the SA group complained of numbness and immobility of the lower limbs during and early after surgery, which resulted in their discomfort. The maximum sensory blockade segments were more extensive in the EAC and SA groups than in the EA group. A denser lower limb motor block and a higher incidence of intraoperative maternal hypotension were observed in the SA group. Patients and the obstetrician were more significantly satisfied with the intraoperative anaesthesia quality in the EAC group than in the EA group. This result indicated that epidural anaesthesia combined with caudal anaesthesia may be a better choice for selective caesarean section.

A previous clinical study found that postoperative satisfaction was lower in parturients receiving spinal anaesthesia than in parturients receiving epidural anaesthesia [15]. In our study, compared to patients undergoing single-space epidural anaesthesia or epidural anaesthesia combined with caudal anaesthesia, the patients who received spinal anaesthesia had lower postoperative comfort, which was consistent with the above findings. Postdural puncture headache is a well-known iatrogenic complication of neuraxial anaesthesia [16]. A lower incidence of PDPH could improve maternal satisfaction [17]. In the present study, PDPH occurred in one patient with accidental dural puncture in each of the EAC and EA groups, whereas no PDPH occurred in other patients in the two groups. This showed that patients without a dural puncture undergoing epidural anaesthesia would not develop PDPH. Recent studies have reported that the rates of PDPH after spinal anaesthesia were 5.6 and 17.2% [18, 19]. A meta-analysis showed that the occurrence of PDPH could be reduced by using a traumatic versus conventional needle (4.2% vs. 11%) [20]. This headache typically changes with position, and the patients were advised to remain supine for several hours following the procedure to prevent and reduce the occurrence of PDPH [21]. In our study, we used 25Gpencil-point spinal needles, and the incidence of PDPH in parturients undergoing spinal anaesthesia was 8%, which was lower than the rate of 28% reported by Uluer MS [22].The low incidence of PDPH in our study is mainly due to the strict supine position for 4 hours after the operation in patients undergoing spinal anaesthesia. However, breastfeeding became difficult early after the operation when the parturient remained supine after spinal anaesthesia, which led to a decrease in postoperative maternal comfort.

Numbness of the lower limbs has a greater impact than pain on patient postoperative satisfaction [23]. The local anaesthetic was instilled in the vertebral subarachnoid space during spinal anaesthesia, and the site of local anaesthetic administered was close to the site of action, which leads to a denser sensory block than epidural anaesthesia [24]. Early mobilization after neuraxial anaesthesia is very important for newborn care [25]. In the current clinical trial, more parturients undergoing spinal anaesthesia complained of postoperative numbness and motor weakness in the lower extremities, which made it difficult for them to care for their babies and subsequently reduced postoperative maternal comfort. Some studies have shown that differences in epidural catheter placement sites can lead to different incidences of numbness and motor weakness in the lower extremities [26, 27]. Patients with caudal epidural catheter placement had a higher incidence of numbness and motor weakness in the lower extremities [26]. In our study, the epidural catheter placement site was at the L_2–3_ interspace in the EA group, and a large volume of high-concentration local anaesthetics (0.75% ropivacaine, 16 ml) was administered to the epidural space for epidural anaesthesia in caesarean section. Similar to the spinal anaesthesia group, some parturients in the EA group complained of numbness and motor weakness in the lower extremities. The epidural catheter was placed at the T_11–12_ interspace in the EAC group, and few parturients developed numbness and weakness in the lower limb. This result indicated that the epidural catheter should be placed at a more cephalad position, which could reduce the adverse effects on the lower limb [26].

The blockade level is an important factor in determining adequate intraoperative analgesia during regional blocks in caesarean Section [28].The skin incision of the caesarean section was located between T_10_-T_12_, and the surgical procedures were mainly performed in the pelvic cavity. Moreover, sensory fibres that innervate the body of the uterus and the cervix pass mainly via sympathetic nerves (T_10_–L_1_) and parasympathetic nerves (S_2–4_), respectively [29]. Therefore, a caesarean section anaesthetic segment requires at least from the 10th thoracic nerve to the sacral nerve, and cesarean section requires a sensory blockade level up to T_4_ clinically [7]. The local anaesthetic was injected into the vertebral subarachnoid space during spinal anaesthesia, and the administration site is close proximity to the action site of local anesthetic. This leads to a shorter duration of local anesthetic action, faster onset, smaller drug dosage, and more precise block in spinal anaesthesia, and being more popular for caesarean section compared to epidural anaesthesia [30, 31]. In the present study, we found that the time to cryanaesthesia at T_10_ and time to maximum motor block were shorter in the SA group, and better muscle relaxation was judged by the obstetrician during spinal anaesthesia, which is similar to the results described for women receiving spinal anaesthesia for caesarean section, who showed that the time from anaesthesia to the start of the surgery was reduced [32]. The maximal segments of sensory blockade were fewer in the EA group than in the SA and EAC groups, and inadequate epidural anaesthesia in six patients was converted to general anaesthesia, while no conversion to general anaesthesia was observed in the EAC and SA groups. It was suggested that it was difficult for single-point epidural anaesthesia to achieve such a wide range of spinal nerve blocks to proceed with the operation. To avoid incomplete anaesthesia during epidural anaesthesia, a large volume of local anaesthetic (26–35 ml) was injected into the epidural space for the spread of anaesthesia [33]. However, if the volume is increased without reducing the local anaesthetic concentration, it is very likely to cause systemic toxicity. High-concentration local anaesthetics can block nerve impulse conduction in both motor nerves and sensory nerves, and low-concentration local anaesthetics can only block impulse conduction in sensory nerves [34].During caesarean section, obstetricians required the relaxation of maternal abdominal muscles and little requirement for relaxation of pelvic floor muscles. Therefore, a high concentration of local anaesthetic (0.75% ropivacaine) was administered to the epidural space of T_11–12_ for the relaxation of maternal abdominal muscles and blocking visceral sensory, and a low concentration of local anaesthetic (0.225% ropivacaine) was injected into the sacral canal for visceral pelvic pain in this study. Although the total volume of local anaesthetics was 30 ml in the EAC group, the dosage of local anaesthetic was not increased correspondingly, which was the same as that of single point epidural anaesthesia. Segments of spinal blockade were more significantly increased in the EAC group than in the EA group, and the degree of abdominal muscle relaxation evaluated by obstetricians during the operation was as good as that in spinal anaesthesia. This showed that caudal anaesthesia with a low concentration of local anaesthetic can significantly improve the quality of epidural anaesthesia during caesarean section.

Hypotension is the most common problem associated with neuraxial anaesthesia, and treatment for hypotension is more likely when spinal anaesthesia is used [35, 36]. The incidence of hypotension during spinal anaesthesia for caesarean section varies from 53 to 85%worldwide [37]. The possible mechanism of subarachnoid block-induced hypotension is related to spinal nerve sympathectomy, vasodilation of peripheral arteries, decreased venous reflux, and consequently decreased cardiac output [38]. According to the current study findings, the incidence of hypotension was 44% in the SA group. The incidence of hypotension in our study was lower than those reported, which was perhaps due to the pre-anesthesia infusion of 500 ml sodium chloride solution, left uterine displacement in parturients, and the level of the blockade being less than T_4_ in spinal anaesthesia. However, the incidence of hypotension was higher in the SA group than in the EA and EAC groups (the incidences of hypotension in the EA and EAC groups were 20 and 22%, respectively). It seemed that the haemodynamics of parturients were more stable in epidural anaesthesia and epidural anaesthesia combined with caudal anaesthesia than in spinal anaesthesia. The hypotension during spinal anaesthesia can cause parturients dizziness, nausea and vomiting, which also led to lower satisfaction in the SA group than in the EAC group.

There were several limitations in our study. First, none of the patients in this study underwent ultrasound examinations, and the postoperative thromboembolic events were unclear. In caesarean delivery women, maternal death and maternal morbidity caused by PE are more common [39]. A higher risk of postoperative venous thromboembolism was associated with spinal anaesthesia than with epidural anaesthesia [40]. In our study, we required parturient to actively turn over after cesarean section to promote accelerated blood circulation. If the patients develop a hypercoagulable state, anticoagulants were administered accordingly. Second, we did not investigate the effects of spinal anaesthesia, epidural anaesthesia and epidural anaesthesia combined with caudal anaesthesia on breastfeeding during the early postpartum period. Infants who initiated breastfeeding within an hour of birth had a 33% lower risk of neonatal mortality compared to infants who initiated breastfeeding between 2 and 23 hours after birth [41]. Some parturients who received subarachnoid block complained about being put in the supine position for at least 4 hours after surgery and being unable to sit up for breastfeeding and foetal care during this time, which may have a negative effect on the infants. We guided the patient’s relatives to assist the mother in early breastfeeding, and all fetuses were promptly breastfed early after surgery. Third, motor blockade decreased with decreasing concentrations of local anaesthetic [42]. In our study, 0.225% ropivacaine injected into the sacral canal significantly improved the quality of epidural anaesthesia during caesarean section. However, the optimal effective concentration of ropivacaine for caudal anaesthesia during caesarean section has not been established. Fourth, we did not perform epidural punctures at L_2–3_ interspaces in the EAC group; thus, the effect of epidural anaesthesia (L_2–3_) combined with caudal anaesthesia for caesarean section was not clear. In order to minimize the lower limb motor block caused by higher concentrations of local anesthetics and increase the block segment without increasing the dose of local anesthetics, we performed epidural anesthesia at T_11_-T_12_ interspace in EAC group instead of L_2–3_ interspace.

In conclusion, epidural anaesthesia combined with caudal anaesthesia may be a better choice for elective caesarean section with a higher quality of intraoperative anaesthesia and postoperative comfort. It is easy to perform by ultrasound, maternal haemodynamics are more stable, and the incidence of complications is lower.

## Data Availability

The identified datasets analyzed during the current study are available from the corresponding author upon reasonable request.
